# Combining Radiotherapy and Immunotherapy in Lung Cancer: Can We Expect Limitations Due to Altered Normal Tissue Toxicity?

**DOI:** 10.3390/ijms20010024

**Published:** 2018-12-21

**Authors:** Florian Wirsdörfer, Simone de Leve, Verena Jendrossek

**Affiliations:** Institute of Cell Biology (Cancer Research), University Hospital Essen, 45147 Essen, Germany; florian.wirsdoerfer@uk-essen.de (F.W.); simone.deleve@uk-essen.de (S.d.L.)

**Keywords:** Irradiation, immune checkpoint inhibition, pneumonitis, fibrosis, T cells, pneumopathy, adverse effects, PD1, PD-L1, CTLA-4

## Abstract

In recent decades, technical advances in surgery and radiotherapy, as well as breakthroughs in the knowledge on cancer biology, have helped to substantially improve the standard of cancer care with respect to overall response rates, progression-free survival, and the quality of life of cancer patients. In this context, immunotherapy is thought to have revolutionized the standard of care for cancer patients in the long term. For example, immunotherapy approaches such as immune checkpoint blockade are currently increasingly being used in cancer treatment, either alone or in combination with chemotherapy or radiotherapy, and there is hope from the first clinical trials that the appropriate integration of immunotherapy into standard care will raise the success rates of cancer therapy to a new level. Nevertheless, successful cancer therapy remains a major challenge, particularly in tumors with either pronounced resistance to chemotherapy and radiation treatment, a high risk of normal tissue complications, or both, as in lung cancer. Chemotherapy, radiotherapy and immunotherapy have the capacity to evoke adverse effects in normal tissues when administered alone. However, therapy concepts are usually highly complex, and it is still not clear if combining immunotherapy with radio(chemo)therapy will increase the risk of normal tissue complications, in particular since normal tissue toxicity induced by chemotherapy and radiotherapy can involve immunologic processes. Unfortunately, no reliable biomarkers are available so far that are suited to predict the unique normal tissue sensitivity of a given patient to a given treatment. Consequently, clinical trials combining radiotherapy and immunotherapy are attracting major attention, not only regarding efficacy, but also with regard to safety. In the present review, we summarize the current knowledge of radiation-induced and immunotherapy-induced effects in tumor and normal tissue of the lung, and discuss the potential limitations of combined radio-immunotherapy in lung cancer with a focus on the suspected risk for enhanced acute and chronic normal tissue toxicity.

## 1. Introduction

Radiotherapy is part of standard care for cancer patients. More than 60% of all cancer patients receive radiotherapy alone or in multimodal combinations of surgery, radiotherapy and chemotherapy during the course of their disease, resulting in favorable effects on local tumor regression, long-term survival, and even tumor cure [1,2,3,4]. However, treatment outcome still needs to be improved for cancer types with high loco-regional failure rates, a high risk for invasive growth or metastatic spread, or a high risk for normal tissue complications, such as non-small cell lung cancer (NSCLC).

It is widely known that tumor cell intrinsic factors, such as resistance-promoting mutations and tumor cell plasticity, as well as a pronounced tumor heterogeneity, a resistance-promoting microenvironment, and the pronounced radiosensitivity of the normal lung tissue, limit the efficacy of radiotherapy to the thoracic region or concurrent platinum-based radiochemotherapy [5,6]. Advances in image-guided radiotherapy and the use of novel radiation techniques such as intensity-modulated radiotherapy and proton therapy have improved the accuracy and safety of thoracic radiotherapy [7,8,9], but the risk for normal tissue complications still limits the application of curative radiation doses to the thoracic region, whereas tolerable doses might be linked to loco-regional failure, despite accepting adverse effects that reduce the quality of life [10,11]. Thus, new and biologically optimized strategies for the radiotherapy of lung cancer with acceptable safety profiles and durable responses are needed to overcome these limitations.

In an effort to identify novel and effective treatment strategies to combat cancer, in recent decades, researchers have put a lot of effort into unraveling the principles of the physiological immune response against cancer and the mechanisms of tumor immune escape. These studies revealed, among other things, that an efficient immune response against cancer requires the activation of tumor-specific CD8^+^ T cells directed against tumor-associated antigens [12,13], and that patients with advanced solid tumors either present tumors with evidence (“hot” immune-sensitive tumors) or without evidence (“cold” immune-resistant tumors) for a T-cell inflamed tumor microenvironment [14,15]. Moreover, it was discovered that, apart from increased production of immunosuppressive mediators such as *transforming growth factor beta* (TGF-β) or the propagation of regulatory T cells (Treg), tumor cells and immune cells up-regulate specific proteins on their surface, namely c*ytotoxic T-lymphocyte-associated Protein 4* (CTLA-4), *programmed cell death protein 1* (PD1), or *indoleamine 2,3-dioxygenase* (IDO) on immune cells, and *programmed cell death 1 ligand 1* (PD-L1), as well as CTLA-4 and IDO on tumor cells, that enable tumor immune escape in tumors with an initial immune response [16,17,18,19,20]. These findings resulted in the development of several therapeutic strategies aimed at the (re)activation of the antitumor immune responses in cancer patients. Nowadays, immunotherapies, particularly immune checkpoint inhibition (ICI) of CTLA4 and PD1/PDL1, are increasingly used as a promising and effective systemic cancer treatment, boosting the immune response, and thus leading to successful immune recognition and tumor cell killing [21,22,23]. However, only a fraction of patients is sensitive to ICI treatment (responders), some patients fail to ever respond (innate resistance), and some patients even develop therapy resistance after a short initial response phase (acquired resistance) [24,25]; moreover, patients may suffer from immune-related adverse effects [26]. Thus, further work is necessary to increase the efficacy of immunotherapy by optimal combinations with other immunotherapy approaches, or cytotoxic chemotherapy or radiotherapy.

The use of radiotherapy as a standard treatment option in the therapy of solid human tumors is based on its ability to locally damage cellular macromolecules, particularly DNA. Thereby, exposure to ionizing radiation effectively induces growth arrest and cell death in irradiated tumor cells, resulting in tumor shrinkage and potentially in tumor elimination. However, the discovery that radiation-induced damage to tumor tissues and normal tissues in the radiation field can trigger the activation of the immune system via well-known damage-signaling cascades, immunogenic cell death, or both, has led to a paradigm change in the use of radiotherapy. Preclinical and clinical investigations revealed a complex interplay between radiotherapy, irradiated cells and tissues, and the immune system; for example, exposure to radiotherapy was shown to up-regulate *major histocompatibility complex I* (MHCI) expression in tumor cells, modulate immunosuppressive barriers in the tumor microenvironment, activate restrictive tumor vessels, trigger the recruitment of immune effector cells to the local tumor, and even elicit systemic tumor-specific immune responses leading to the regression of tumor nodules outside the radiation field (abscopal effects) [27,28,29]. However, such abscopal responses to radiotherapy alone are only occasionally observed in patients, presumably because the tumor microenvironment efficiently shapes tumor immune escape at multiple levels and thus hampers a beneficial radiation-induced immune activation [30,31]. Because of the limited success of conventional therapies in patients with resistant and metastatic tumors, current clinical studies focus on combining radiotherapy with immunotherapy, particularly ICI, to overcome these limitations and harness the combined therapeutic potential of both therapies. The first data of such studies demonstrate that blockade of the PD-1/PD-L1 immune checkpoint improves progression-free survival in a fraction of NSCLC patients with an acceptable safety profile when given after radiotherapy or platinum-based radiochemotherapy [32,33]. Moreover, radiotherapy and CTLA-4 blockade were effective in inducing a systemic anti-tumor T cell response in chemo-refractory metastatic NSCLC that failed to respond to anti-CTLA-4 antibodies alone or in combination with chemotherapy [34]. This study also revealed a rapid expansion of CD8^+^ T cells recognizing a neoantigen encoded by a radiation-induced gene, thereby pointing to a contribution of radiation-induced exposure of immunogenic factors to the systemic antitumor response.

There is hope that the use of T cell-stimulatory immunotherapies or small-molecule inhibitors of immunosuppressive signaling pathways in the tumor microenvironment in combination with surgery, radiotherapy and/or chemotherapy will revolutionize the success of standard of care for cancer patients in the long term. However, radiotherapy can also up-regulate immunosuppressive signaling cascades in the tumor microenvironment, such as TGF-β signaling or expression of PDL1. Moreover, radiation-induced immune activation participates in inflammation-associated normal tissue responses as well as severe, potentially life-threatening acute and chronic inflammatory adverse effects upon thoracic or total body irradiation such as pneumonitis and pulmonary fibrosis [35]. Thus, interfering with the immune system by multiple, consecutive therapeutic interventions with immune activating effects, such as radio(chemo)therapy and immunotherapy, might increase the risk of deregulated and unwanted adverse (auto)immune responses even when given after a temporal gap. For example, a recent case report where radiotherapy was followed by ICI treatment revealed severe inflammatory side effects [36]. The observed inflammatory tissue toxicities suggest potential limitations for combined radio(chemo)therapy and ICI treatment with respect to long-term safety.

Several recent reviews discussed the potential of combining immunotherapy and radiotherapy to treat lung cancer with respect to clinical efficacy [37,38,39,40,41,42,43,44,45]. Therefore, here we will only briefly summarize the current knowledge of tumor-induced immune escape and the contribution of radiotherapy in modulating tumor immune responses. Instead, we will discuss in more detail potential risks and limitations that may occur when combining radiotherapy and immunotherapy in lung cancer with a focus on the effects of radiation-induced immunomodulation for suppression of efficient anti-tumor immune responses and normal tissue toxicity in the lung.

## 2. Radiotherapy in the Thoracic Region—Tumor Control Versus Immune Escape

Lung cancer currently represents the second most common cancer, as well as the leading cause of death in men and women in the United States (National Cancer Institute, available online: https://seer.cancer.gov) [46]. It is also one of the most common cancers among both men and women in Germany; the 1st in men (25% of deaths due to cancer), and the 2nd in women (15% of deaths due to cancer) (German Centre for Cancer Registry Data, available online: https://www.krebsdaten.de). The majority of cases comprise localized and locally advanced non-small cell lung cancer (NSCLC). Patients are typically treated with surgical resection, radiotherapy and chemotherapy. Stereotactic body radiation therapy (SBRT) is frequently used as an effective treatment in patients with inoperable or early-stage tumors, whereas inoperable patients with locally advanced tumors receive concurrent chemoradiation with conventional fractionated radiotherapy [47,48,49]. The results and outcomes from clinical trials of the different treatment regimens in lung cancer have been summarized elsewhere [37,49,50,51,52,53]. Thus, we will focus in this part of the review on how exposure to ionizing radiation shapes the immune response towards tumor control and the tumor microenvironment towards immune escape, respectively.

Radiation-induced alterations in the tumor microenvironment involve the direct activation of innate and adaptive immune cells with various effects on tumor growth and tumor cell killing, but also indirect changes in the tumor microenvironment and the tumor vasculature that alter the recruitment and activation state of cells from the innate and adaptive immune system (for a review, see [54,55,56,57,58,59,60]).

Radiotherapy induces damage and cell death in cancer cells, leading to the exposure of immunogenic molecules such as calreticulin on the cell surface [61]. Moreover, damage-associated molecular patterns (DAMPs), such as uric acid, S100 proteins, *adenosine triphosphate* (ATP) or *High-Mobility-Group-Protein B1* (HMGB1), as well as tumor antigens, are released to activate innate and adaptive immune responses [62,63]. In addition, radiation-induced nuclear DNA release and subsequent sensing of cytoplasmic dsDNA can activate the c*GMP–AMP synthase* (cGAS)–*stimulator of interferon genes* (STING) pathway, which is closely related to the activation of a type I interferon response with expression of inflammatory genes and the secretion of cytokines that promote antitumor immunity. Uptake of tumor antigens by antigen presenting cells, e.g., dendritic cells, as well as sensing of cancer cell-derived cytoplasmic dsDNA by the cGAS/STING pathway are required for the priming of tumor-specific T cell responses. To trigger the activation of B and T cells, mature dendritic cells migrate to and present antigens in secondary lymphoid organs. Activated T cells and B cells can exert systemic anti-tumor effects by several mechanisms like CD8^+^ T cell mediated cytotoxicity, antibody-dependent cell-mediated cytotoxicity, and antibody-induced complement-mediated lysis [56,61,62,64,65,66,67,68].

A complex interaction between the tumor microenvironment and the immune system is needed to achieve a radiation-induced anti-tumor immunity. Moreover, the induction of tumor antigen-presenting dendritic cells that are essential for T cell priming largely depends on the radiation dose and fractionation in a tumor-dependent manner. For example, the radiation dose per fraction dictates the level of the exonuclease *three prime repair exonuclease 1* (TREX1), which degrades interferon-stimulatory cytosolic dsDNA and thus abrogates the immunogenicity of irradiated cancer cells [67,69,70]. Finally, tumor cells exert a range of acquired capabilities to escape the immune system, so that direct anti-tumor immunity in response to radiotherapy alone is a rare event [30,67,71].

Besides radiation-induced anti-tumor immune responses, tumor irradiation can also activate immune cells with tumor-promoting properties. Pro-inflammatory cytokines that are released after radiation-induced tissue damage, as well as the humoral immune response from activated B cells, trigger the recruitment and activation of innate immune cells, such as granulocytes, macrophages and mast cells [72,73]. By releasing several mediators, these innate immune cells can alter gene expression programs, thus favoring pro-survival signaling and cell cycle progression, as well as tissue expansion in tumors [74,75]. Moreover, innate immune cells can release various mediators, which have an impact on fibroblast activation, angiogenesis, and matrix metabolism. Innate immune cells thus have the ability to induce repair, regeneration and tissue remodeling in favor of tumor growth [73,76,77,78].

**Figure 1:**
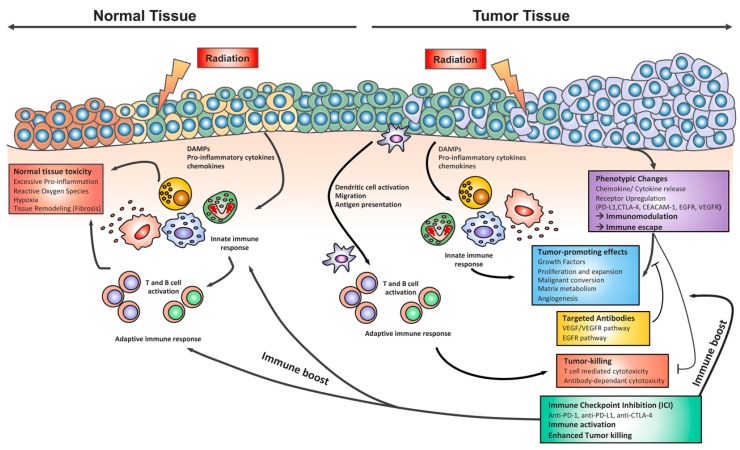
Radiotherapy of malignancies in the thoracic region results in stress, damage and cell death in the tumor tissue, and to some extent also in surrounding normal tissue within the radiation field. The release of damage-associated molecular patterns (DAMPs), cytokines and chemokines leads to the recruitment of immune cells to the site of damage. In the normal lung tissue, the influx of pro-inflammatory immune cells can induce an acute normal tissue-toxicity (pneumonitis) with excessive inflammation, or on the long-term, chronic inflammation, environmental changes and tissue remodeling (fibrosis). In contrast to that, the innate immune system contributes to tumor progression due to tumor-promoting activities. Radiation-induced cell death of tumor cells also results in the release of tumor antigens to activate dendritic cells with subsequent antigen presentation and induction of T cell-mediated tumor killing, a process termed “in situ vaccination”. However, several tumor-intrinsic mechanisms shape the innate immune response towards tumor promotion and adaptive immune responses towards immunosuppression, so that the tumor cells escape from T cell-mediated cytotoxicity. The use of immune checkpoint inhibition (ICI) boosts the immune system, overcoming immunosuppression and immune escape, and thus leading to successful eradication of tumor cells. In normal tissues, ICI might trigger an unbalanced immune activation, thereby potentially enhancing normal tissue toxicity. Combined treatment with ICI and radiotherapy bears the potential risk of synergistic toxic effects, depending on several variables in the treatment setting of radioimmunotherapy.

Finally, radiation-induced stress or damage in the tumor itself results in several phenotypic changes. By secreting cytokines, chemokines or growth factors, as well as up-regulating specific surface receptors, e.g., PD-L1, CTLA-4, *carcinoembryonic antigen-related cell adhesion molecule 1* (CEACAM1), and others, tumor cells become competent in dampening and finally escaping the immune system (Figure 1) [79,80,81,82,83,84]. Several therapeutic strategies aim at the inhibition of such tumor cell-extrinsic factors that lead to primary and adaptive resistance and will be addressed in more detail in the “Immunotherapy in lung cancer” section, but have also been nicely reviewed by others [24,30].

## 3. Radiation-Induced Normal Tissue Toxicity in the Lung

The lung, with its mucosal barrier, is constantly exposed to foreign particles and pathogens. Therefore, the mucosal barrier is equipped with means to recognize and eliminate any harmful exposures, thereby functioning as an immunological organ. Epithelial cells, alveolar macrophages, and other immune cells in the respiratory tract express pathogen recognition receptors (PRR) to direct powerful immune responses if needed, for example, during infection, leading to immune cell recruitment and inflammation [85]. Besides the recognition of foreign harm, the same PRR can identify endogenous danger signals; the so-called DAMPs.

Despite technological improvements, radiation still directly hits, to some extent, tumor-surrounding healthy tissues during treatment, e.g., the highly radiosensitive lung tissue. Radiation-induced stress, damage or cell death to lung resident cells and local immune cells triggers the release of several DAMPs into the extracellular room, immunogenic cell death, or both. Through recognition of these DAMPs via PRR, the radiation-induced damage induces a sterile inflammation in sensitive patients that can evolve into a harmful normal tissue inflammation with life-threatening complications [86,87]. It is noteworthy that the damage response from the malignant and healthy tissue residing in the radiation field not only contributes to the local and abscopal effects of radiotherapy against the tumor, but can also induce strong systemic side effects [88,89,90,91,92]. Moreover, as described before, radiation-induced DNA damage and subsequent detection of cytosolic DNA can activate the cGAS/Sting pathway, which is important for the activation of an innate and adaptive antitumor immunity [93,94].

Briefly, the released “danger signals” induce a subsequent chemokine/cytokine secretion, innate and adaptive immune cell recruitment, and activation of recruited immune cells at the site of radiation damage leading to tissue inflammation. This inflammatory response is needed to orchestrate tissue repair and regeneration in order to restore tissue homeostasis. If the inflammation during the acute phase is too excessive, due to overwhelming release of cytokines and reactive oxygen species (ROS), the inflammation can exert toxic effects in the normal tissue (pneumonitis), a severe side effect in patients [95]. Radiation-induced pneumonitis can develop at 4 to 12 weeks after radiotherapy, with symptoms including fever, dry cough, chest pain and dyspnea, or even failure of the respiratory system. The reported occurrence of pneumonitis in lung or breast cancer patients ranges from 13 to 36% of patients, depending on the diagnosed severity [34,96]. Patients suffering from severe radiation-induced pneumonitis are commonly treated with anti-inflammatory corticosteroids, such as prednisone [35,96,97,98,99]. 

Besides radiation-induced acute inflammatory responses, subsequent repair/regeneration processes can manifest as a chronic event, where pathologic immunomodulation and altered microenvironmental changes, e.g., hypoxia, senescence and anti-inflammation, drive excessive tissue remodeling (fibrosis); moreover, exposure to ionizing radiation can also increase the risk for secondary tumor formation on the long-term [57,87,92,100,101,102,103,104,105,106,107]. Radiation-induced lung fibrosis mostly develops 6 to 24 months after radiotherapy, and is characterized by breathing difficulties and subsequent volume loss of the lung [35,108]. The mechanisms of radiation-induced lung disease have been described in detail elsewhere and will not be addressed here [35,92,106,109].

Both the acute and the chronic events of radiation-induced lung disease are accompanied by microenvironmental alterations in the lung tissue, as well as local and systemic immune changes. Current research focuses on the identification of target cells, pathologic signaling molecules and up-stream regulators to develop novel strategies for prevention or treatment against these adverse effects. We speculate that tumor-induced and radiation-induced immune changes in tumor and normal tissues might offer the opportunity to improve immune-mediated tumor killing and to counteract at the same time radiation-induced chronic adverse effects in the lung tissue [110,111].

This is particularly interesting, as the first promising results have recently been obtained using immunotherapy with ICI in combination with radiotherapy for the treatment of NSCLC [32,33,34]. However, as outlined above, radiotherapy modulates the immune repertoire in tumor tissues and normal lung tissue in various ways. Thus, optimal targeting of the immune system in combination with radiotherapy will require further work to define therapeutic strategies that balance pro-immunogenic and immunosuppressive effects of radiotherapy and outweigh the beneficial effects of radioimmunotherapy between optimal tumor control and normal tissue protection.

## 4. Immunotherapy in Lung Cancer

As mentioned in the sections above, tumors develop intrinsic or radiation-induced alterations that shape an immune phenotype with immunosuppressive or even tumor-promoting characteristics. Major receptors or pathways involved in immunomodulation include *vascular endothelial growth factor receptor* (VEGFR), *epidermal growth factor receptor* (EGFR), as well as PD-L1 on tumor cells and the receptors PD1 and CTLA-4 on the site of T cells [112]. However, CTLA-4 expression was also detected on multiple tumor cells including NSCLC [19]. Interestingly, a recent study revealed that tumor cell-intrinsic CTLA-4 expression had an impact on the expression of PD-L1, as well as on tumor cell proliferation. Inhibition of CTLA-4 induced the upregulation of PD-L1, as well as the activation of the EGFR pathway in NSCLC cells, highlighting a distinct function of CTLA-4 in tumor cells compared to T cells [113].

Generally, surface expression of the receptors VEGFR and EGFR on tumor cells and their interaction with the corresponding growth factors induces tumor-promoting proliferation, expansion, and malignant conversion of cancer cells, as well as tumor-promoting angiogenesis. VEGF and EGF are commonly released by innate immune cells, but tumor cells can also secrete VEGF in an autocrine manner, thereby stimulating cancer stemness [114,115,116]. Generally, VEGF is a proangiogenic growth factor and potent inducer of growth and survival of the vascular endothelium. Besides its direct effect on the tumor vasculature, VEGF can also modulate the immune environment [117]. Several studies demonstrated that VEGF has the ability to inhibit the differentiation and function of diverse immune cells like dendritic cells, macrophages, and lymphocytes, respectively [118,119,120]. More details about the role of VEGF/VEGFR in tumor and immune cells can be found in the following reviews: [121,122]. Activation of EGFR on tumor cells, including NSCLC, also promotes proliferation, invasion, metastasis, anti-apoptotic signaling and angiogenesis in malignant tumors [123,124,125]. Lung cancer patients have intrinsic or acquire new EGFR mutations during treatment with small-molecule EGFR-inhibitors that complicate current treatment options highlighting the need for the development of novel strategies for EGFR-resistant NSCLC, including combined therapies with ICI or radiotherapy [10,126,127,128,129].

In contrast to VEGF and EGF, PD1/PD-L1, as well as CTLA-4 are immune checkpoints that negatively regulate T-cell immune functions, thus indirectly promoting tumor progression via tumor immune escape [130,131,132]. Our understanding of the mechanisms and pathways how cancers evade and inhibit immune responses and their targeting has largely improved over the years and the state of knowledge has been summarized in recent reviews [23,133,134,135,136]. Overall, targeting these structures or pathways with immunotherapies, alone or in combination, substantially improved the care of patients with advanced-stage cancers leading to longer survival rates or even long-lasting tumor remissions. Several of these immunotherapeutic strategies are being evaluated in lung cancer [137,138,139,140,141]. Multiple strategies using either T cell-stimulating agents (*tumor necrosis factor receptor* (TNFR) superfamily antibodies and peptides, e.g., OX-40), genetically modified T cells (*Chimeric Antigen Receptor* (CAR)-T cells), bi-specific antibodies, and tumor vaccination or agents that counteract the immunosuppressive tumor microenvironment (e.g., inhibitors of TGF-β, as well as Toll-like receptor (TLR) agonists) are currently being tested in preclinical and clinical trials [141,142,143,144,145,146,147,148,149,150].

So far, two groups of targeted compounds are FDA approved; these include, on the one hand, “targeted antibodies” against the VEGF/VEGFR pathway (Bevacizumab), and the EGFR pathway (Necitumumab), which are being used in a subset of patients with advanced NSCLC [151,152]. Both target the tumor-promoting effects of growth-factor-induced proliferation, expansion and angiogenesis. On the other hand, ICI have been approved by the FDA for cancer treatment, and so far, they have received the most clinical recognition among the current immunotherapeutic agents. ICIs have emerged as a landmark event for the treatment of lung cancer; among the FDA-approved ICIs, Pembrolizumab and Nivolumab (both anti-PD1), as well as Atezolizumab and Durvalumab (both anti-PD-L1), and Ipilimumab (anti-CTLA-4), alone or in combination with each other, were effective against *small cell lung cancer* (SCLC), NSCLC, and metastatic lung carcinomas, and improved the prognosis in these patients [153,154,155,156,157,158].

Despite the revolutionary efficacy profile of ICIs, which has raised cancer therapy to a new level, it has to be taken into consideration that unbalancing the immune system with ICI can also generate adverse events with severe complications in patients [159]. These so-called *immune-related adverse effects* (IRAEs) can occur in the gut, skin, endocrine glands, liver, heart and lung, but can potentially involve any tissue [160,161,162,163,164,165,166]. Increasing the knowledge gained over the last years in several trials with ICI has made oncologists aware of the potential risks, but not all variables are yet understood, e.g., patient predisposition or pretreatment status of the patient. Several recent reviews highlight the potential risks that could emerge using ICI alone [167,168,169,170,171,172].

In brief, an ICI-induced immune dysregulation towards an “immune boost” with uncontrolled pro-inflammatory signaling can trigger toxic effects and normal tissue complications. It is already known, e.g., from trauma patients that tissue damage can unbalance the immune system with overwhelming pro-inflammatory responses and the induction of a *systemic inflammatory response syndrome* (SIRS). Due to this, severe toxic side effects, multiple organ failure, or even death can occur in these patients [173]. Therefore, it is not surprising that ICI can also induce SIRS in cancer patients, including lung cancer [174]. It has to be considered that irradiation also induces tissue damage (trauma) and inflammation with the potential to induce normal tissue toxicity. Similar to trauma patients, the extent of induced tissue-damage in irradiated patients will be critical for the extent of the immune response. Knowing that immunotherapy alone can already induce IRAEs in several tissue types, including the lung, pre- or post-treatment with radio(chemo)therapy might have additive or synergistic life-threating effects in patients that are already fragile in health.

## 5. Combining Radiotherapy and Immunotherapy in Lung Cancer

Several recent reviews have discussed the potential of combining immunotherapy and radiotherapy, including for lung cancer, with respect to clinical efficacy [37,38,39,40,41,42,43,44,45]. The recent review from Ko et al. published in June 2018 nicely summarizes planned and ongoing trials of combined radioimmunotherapy in patients with NSCLC with respect to present knowledge [37]. Those trials include phase 1 and 2 studies, with disease stages ranging from early stage NSCLC and locally advanced stage NSCLC towards the majority of trials investigating stage IV metastatic NSCLC. Among these trials, all potential settings are under investigation, including the use of different inhibitors and molecules (durvalumab, atezolizumab, pembrolizumab, tremelimumab, nivolumab, ipilimumab, *granulocyte-macrophage colony-stimulating factor* (GM-CSF), thymosin-α1, as well as virus therapy), different combined inhibitor administrations, and different timing and dosing of radioimmunotherapy. Thus, ongoing clinical trials will add important knowledge and evidence related to this novel and promising combinatory cancer therapy.

The majority of reviews highlight the two comprehensive trials, namely the phase I KEYNOTE-001 trial of pembrolizumab (anti-PD1) in patients with metastatic NSCLC [32] and the phase III PACIFIC trial of durvalumab (anti-PD-L1) in patients with unresectable stage III NSCLC after ≥2 cycles of platinum-based chemoradiation [33].

Both studies have in common that immunotherapy was administered subsequent to radiotherapy. The KEYNOTE-001 trial is a secondary analysis of 97 patients with 24 patients that received thoracic radiotherapy at a median of 11.5 months prior to ICI. Overall pulmonary toxicity was observed in 63% of all patients that received prior radiotherapy versus 40% in patients that received no radiotherapy. The incidence of all-grade ICI-related pulmonary toxicities was significantly higher (13% versus 1%, *p* = 0.046) in patients with previous radiotherapy treatment. Although significant changes were observed, the study revealed that there are no differences in high-grade (≥3) pulmonary toxicities between the radiotherapy and non-radiotherapy group (4% versus 1%, *p* = 0.44).

In the PACIFIC trial, 713 patients were analyzed after sequential ICI treatment starting between 1–42 days after chemoradiation. In the combined radiotherapy/ICI group all-grade pneumonitis was more frequent than in the radiotherapy alone group (33.9% versus 24.8%, no *p*-Value). Nevertheless, the authors reported that the incidence of grade ≥3 pneumonitis was similar in both groups (3.4% versus 2.6%, no *p*-value).

Overall, both studies revealed that the risk to develop high-grade pulmonary toxicities is not enhanced when combining radiotherapy and immunotherapy at least with durvalumab and pembrolizumab. What has to be considered is that in the KEYNOTE-001 trial the median interval between radiotherapy and ICI was nearly 1 year. Radiation-induced immunomodulation and pathologic events in normal pulmonary tissues after 1 year are usually described as chronic inflammation with profibrotic tissue remodeling. It might thus not be surprising that ICI does not induce high-grade toxicities after that long-time interval. 

In contrast, in the PACIFIC trial ICI was given earlier (1–42 day) after radiochemotherapy. In general, radiation-induced acute inflammatory effects are observed in the lungs of treated patients during this timeframe shortly after radiotherapy. Consequently, boosting the immune system with ICI during the acute phase after radiotherapy might bear a higher risk of enhancing all-grade toxicities in the combined treatment group. This was not the case in the PACIFIC trial, and this is potentially linked to the type of ICI used. Since durvalumab mainly targets PD-L1, present on tumor cells, immune-related effects are potentially more likely to be linked to the tumor microenvironment and, to a lesser extent, to the normal tissue. Instead, targeting PD-1 or CTLA-4 on immune cells might enhance the immune response in both the tumor and also the normal tissues, thereby increasing the risk for more frequent or more severe pulmonary toxicities upon combined use with radiotherapy when compared to a combination of radiotherapy with PD-L1-inhibition.

Two recent reviews about the toxicities of treatment with ICI alone corroborate this hypothesis: Pillai and colleagues compared 23 studies, including 5744 patients with NSCLC, in a systematic analysis to investigate potential differences in the toxicities of monotherapies using PD-1 and PD-L1 inhibitors. In fact, the authors revealed that the overall incidence of adverse effects was comparable between the PD-1 and PD-L1 inhibitors (64% versus 66%, *p* = 0.8), but in patients treated with PD-1 inhibitors IRAEs (16% versus 11%, *p* = 0.07) and pneumonitis (4% versus 2%, *p* = 0.01) increased compared with patients who received PD-L1 inhibitors [175]. In line with these findings, Khungar and colleagues reported in a systemic analysis including 19 trials, that there was a higher incidence of pneumonitis with the use of PD-1 inhibitors compared with PD-L1 inhibitors in a monotherapy in NSCLC patients [176].

Both the KEYNOTE-001 and the PACIFIC trial show excellent results, as inhibition of PD1 and PD-L1 in combination with radiotherapy significantly increased the objective response rate in patients and significantly extended both the median progression free survival, as well as the median time to death or distant metastasis. However, both trials only tested one potential treatment setting. To fully understand the potential risks of IRAEs that might occur using combined radioimmunotherapy, several further aspects have to be considered, as described below.

One important factor might be the treatment sequence. Should ICI and radiotherapy be given concurrently or sequentially, and does the order of administration have an impact on the outcome? Since radiotherapy of the thoracic region can induce time-dependent changes in the normal lung tissue ranging from pro-inflammatory acute effects towards pro-fibrotic chronic side effects, the different potential treatment settings might also induce distinct effects in the normal lung tissue. Two case reports have revealed that a setting where radiotherapy was given after ICI had beneficial effects. Here, the combination immunotherapy followed by radiotherapy had the capacity to influence tumor responses, overcome resistance to ICI, and even induce abscopal effect with reduced distant metastasis in the lung, at least when using a PD1-inhibitor (Nivolumab) [177,178]. Thus, radiotherapy can stimulate immune activation even when immunotherapy has failed.

However, little is known about potential adverse effects in the lung when radiotherapy is delivered together with ICI. Nevertheless, there are single case reports about adverse pulmonary effects upon concomitant use of ICI and radiotherapy. Louvel and colleagues recently reported two patients, both with metastatic melanoma or metastatic colon cancer, where concomitant radiotherapy and PD-1 or PD-L1 blockade induced radiation-pneumonitis [179]. However, the small sample size makes it difficult to draw firm and clear conclusions, and this was also mentioned by the authors. Thus, additional studies are needed to clarify the acute and long-term safety of the different settings of combining radiotherapy and immunotherapy, particularly ICI.

Besides the treatment schedule of ICI plus radiotherapy, the type of inhibitor that is used might also affect the outcome. PD-1 and PD-L1, but also CTLA-4, are differently expressed in tumor cells and in diverse subsets of immune cells. Furthermore, as mentioned above, radiotherapy shapes the immune repertoire and tissue microenvironment in the tumor, as well as in the normal tissue, in a time-dependent, dose-dependent, volume-dependent, and tissue-specific manner. For example, in the lung, the appearance of distinct immune cells and their phenotypes differs in the acute versus chronic phase after irradiation [87,106,111,180,181]. Thus, modulating the immune response by targeting PD-1, PD-L1 or CTLA-4 in lung cancer might cause varying effects in the normal tissue, depending on the time point (acute versus chronic) of application, especially in a setting where ICI is given after radiotherapy of the thoracic region.

Finally, the patient itself, especially the immune status and the characteristics of the tumor, plays an important role. Besides the need for a better understanding how we can optimally combine radio(chemo)therapy and immunotherapy to best harness the combined potential of both to the benefit of the patient, we also need to focus on the immune characteristics of the individual patient. The improved understanding of cancer biology, as well as the identification of predictive and prognostic biomarkers and of potential therapeutic targets, has supported the development of personalized therapies over recent years [182,183,184,185]. Several case reports highlight the diversity of therapeutic responses in different patients, especially when the immune system is involved (individual innate resistance, acquired resistance and normal tissue toxicity). Thus, an individual evaluation of both, the tumor characteristics and the immune status of a given patient and tumor, are required. Furthermore, the diverse therapeutic responses in different patients highlight the importance to develop tools for a reliable prediction of normal tissue toxicity probability in each patient prior to the combined treatment.

Thus, radiotherapy centers should take the opportunity to collect suitable and structured datasets from the current clinical trials including data about adverse effects. The present technology and available tools for radiotherapy-dose-fractionation-volume parameter assessment, together with the available methodology for estimating normal tissue complication probability (NTCP) [186,187,188,189,190,191] may provide a reliable background for quantitatively detecting “more-than-expected” lung damage, which may be related to the ICI combined administration. Such information can be analyzed according to suspected biological markers to provide suitable hints for prospective trials using radiotherapy and ICI.

## 6. Final Remarks

The current trials combining radiotherapy and immunotherapy show promising results. However, only single possible treatment settings have been tested so far. Moreover, as outlined above, the complex effects of combining irradiation and unbalancing the immune system with ICI are not fully understood, and many open questions remain. Figure 1 provides a schematic overview of the radiation-induced immune changes in tumor and normal tissues. In brief, radiotherapy has the potential to induce stress responses and cell death in cancer cells with subsequent induction of a DC/T cell-driven antitumor immunity. Radiation-induced tissue damage also stimulates the influx of innate immune cells with tumor-promoting potential. These processes are reminiscent of immune changes observed during tumorigenesis, where tumors upregulate a repertoire of cell surface receptors and adaptive changes in the tumor microenvironment to escape T cell killing and foster immunosuppression and tumor-promoting effects such as angiogenesis. 

Immunotherapies were initially developed to cope with the processes involved in tumor immune evasion. ICI interferes with and unbalances the immune response, thereby unleashing an immune boost, at least in a fraction of tumor patients. However, unbalancing the immune response can also induce inflammation-induced toxic side effects in normal tissues. Thus, ICI must be considered a “double-edged sword” that needs careful handling. The same holds true for radiotherapy-induced immune changes; radiotherapy exerts its effects by efficient tumor killing, but it can also damage normal lung tissue. The acute and chronic toxic side effects induced in the radiation-sensitive lung tissue are mediated by a complex interaction between resident lung cells, radiation-induced changes in the lung environment, and cells from the innate and adaptive immune system. Considering that radiation-induced lung disease involves time-dependent (acute and chronic) pathologic immune changes in the lung tissue, a combination with ICI might bear the risk of synergistic adverse effects.

Recent clinical trials only represent a small fraction of potential therapeutic settings of combined radiotherapy and immunotherapy. Thus, only additional trials will shed light on the opportunities and risks of combined radioimmunotherapy and will hopefully clarify which of the various potential treatment settings, e.g., (I) concomitant or sequential treatment, (II) chronology of treatment (radiotherapy > ICI, ICI > radiotherapy), (III) the interval between treatments, or (IV) the type of inhibitor used, has the highest probability for an effective and safe treatment of patients with NSCLC.

## Abbreviations

ATP	Adenosine triphosphate
CAR	Chimeric Antigen Receptor
CEACAM-1	Carcinoembryonic antigen-related cell adhesion molecule 1
cGAS	cGMP–AMP synthase
CTLA-4	Cytotoxic T-lymphocyte-associated Protein 4
DAMPs	Damage-associated molecular patterns
EGF	Epidermal growth factor
EGFR	Epidermal growth factor receptor
GM-CSF	Granulocyte-macrophage colony-stimulating factor
HMGB1	High-Mobility-Group-Protein B1
ICI	Immune Checkpoint Inhibition
IRAEs	Immune-related adverse effects
MHCI	Major histocompatibility complex I
NSCLC	Non-small cell lung cancer
NTCP	Normal tissue complication probability
PD1	Programmed cell death protein 1
PD-L1	Programmed cell death 1 ligand 1
PRR	Pathogen recognition receptor
ROS	Reactive oxygen species
RT	Radiotherapy
SBRT	Stereotactic body radiotherapy
SCLC	small cell lung cancer
SIRS	systemic inflammatory response syndrome
STING	stimulator of interferon genes
TGF-β	transforming growth factor beta
TNFR	tumor necrosis factor receptor
TLR	TOLL-like receptor
TREX1	three prime repair exonuclease 1
VEGF	Vascular endothelial growth factor
VEGFR	Vascular endothelial growth factor receptor
